# *In vivo* Characterization of a Selective, Orally Available, and Brain Penetrant Small Molecule GPR139 Agonist

**DOI:** 10.3389/fphar.2019.00273

**Published:** 2019-03-21

**Authors:** James R. Shoblock, Natalie Welty, Ian Fraser, Ryan Wyatt, Brian Lord, Timothy Lovenberg, Changlu Liu, Pascal Bonaventure

**Affiliations:** Janssen Research & Development, LLC, San Diego, CA, United States

**Keywords:** GPR139, habenula, serotonin, dopamine, behavior

## Abstract

Recently, our group along with another demonstrated that GPR139 can be activated by L-phenylalanine (L-Phe) and L-tryptophan (L-Trp) at physiologically relevant concentrations. GPR139 is discretely expressed in brain, with highest expression in medial habenula. Not only are the endogenous ligands catecholamine/serotonin precursors, but GPR139 expressing areas can directly/indirectly regulate the activity of catecholamine/serotonin neurons. Thus, GPR139 appears expressed in an interconnected circuit involved in mood, motivation, and anxiety. The aim of this study was to characterize a selective and brain penetrant GPR139 agonist (JNJ-63533054) in relevant *in vivo* models. JNJ-63533054 was tested for its effect on c-fos activation in the habenula and dorsal striatum. *In vivo* microdialysis experiments were performed in freely moving rats to measure basal levels of serotonin or dopamine (DA) in prefrontal cortex (mPFC) and nucleus accumbens (NAc). Finally, the compound was profiled in behavioral models of anxiety, despair, and anhedonia. The agonist (10–30 mg/kg, p.o.) did not alter c-fos expression in medial habenula or dorsal striatum nor neurotransmitter levels in mPFC or NAc. JNJ-63533054 (10 mg/kg p.o.) produced an anhedonic-like effect on urine sniffing, but had no significant effect in tail suspension, with no interaction with imipramine, no effect on naloxone place aversion, and no effect on learned helplessness. In the marble burying test, the agonist (10 mg/kg p.o.) produced a small anxiolytic-like effect, with no interaction with fluoxetine, and no effect in elevated plus maze (EPM). Despite GPR139 high expression in medial habenula, an area with connections to limbic and catecholaminergic/serotoninergic areas, the GPR139 agonist had no effect on c-fos in medial habenula. It did not alter catecholamine/serotonin levels and had a mostly silent signal in *in vivo* models commonly associated with these pathways. The physiological function of GPR139 remains elusive.

## Introduction

GPR139 is a G-protein coupled receptor (GPCR) exclusively expressed in the brain and pituitary. High expression of GPR139 has been reported in the medial habenula and lateral septum (Liu et al., 2015). The habenula is part of the limbic circuit involved in the regulation of mood and stress, and neurons from the lateral septum have been shown to modulate neuroendocrine and behavioral stress responses (Singewald et al., 2011; Mirrione et al., 2014), as well as motivation and affect (Sheehan et al., 2004). The expression of GPR139 in the paraventricular nucleus, arcuate hypothalamus, and pituitary suggests that it may play a role in the regulation of stress, hormone production/release, growth, and metabolism. Interestingly, protection of primary dopaminergic midbrain neurons by GPR139 activation has been shown *in vitro* (Bayer Andersen et al., 2016).

Based on surrogate agonists, a pharmacophore model was built leading to the hypothesis that L-tryptophan (L-Trp) and L-phenylalanine (L-Phe) were the putative endogenous ligands for GPR139 (Isberg et al., 2014; Nohr et al., 2017a). We provided additional and independent biological and pharmacological evidence to support that L-Trp and L-Phe activate GPR139 (Liu et al., 2015). The *in vitro* affinity and potency values of L-Trp and L-Phe were within the physiological concentration ranges of L-Trp and L-Phe. In addition, chromatography of rat serum, rat brain, and human serum extract confirmed that L-Trp and L-Phe were the only substances detected capable of activating GPR139. We hypothesized that GPR139 might act as a sensor to detect dynamic changes of L-Trp and L-Phe, which are essential amino acids. A recent publication suggested that *in vitro* GPR139 is also activated by adrenocorticotropic hormone (ACTH), α-melanocyte-stimulating hormone (MSH), β-MSH, and shorter fragments of α-MSH (α-MSH_1-9_, α-MSH_1-10_) (Nohr et al., 2017b). But we reexamined these data and our findings did not support that GPR139 is activated by ACTH, α-MSH, and β-MSH at physiologically relevant concentration although we did unravel an *in vitro* interaction between GPR139 and the melanocortin receptors (Nepomuceno et al., 2018). The signal transduction pathway of GPR139 receptor is not entirely clear but coupling to Gq and calcium mobilization are the most accepted pathways.

Several surrogate small molecule agonists for GPR139 are available (Hu et al., 2009; Shi et al., 2011; Wang et al., 2015; Shehata et al., 2016). Noteworthily, there is a shortage of potent and selective GPR139 antagonists. We disclosed JNJ-63533054 (3-chloro-*N*-[2-oxo-2-[[(1S)-1-phenylethyl]amino] ethyl] benzamide), a new selective brain penetrant GPR139 agonist (Dvorak et al., 2015; Liu et al., 2015). We found that administration of JNJ-63533054 in rats decreased locomotor activity (LMA). Here we provide a more extensive *in vivo* characterization of JNJ-63533054.

*In vitro*, JNJ-63533054 has an EC_50_ of ∼16 nM at human hGPR139, with similar functional potency/affinity at mouse and rat GPR139 (Liu et al., 2015), and was selective for GPR139 against a panel of 50 known other targets (Dvorak et al., 2015). Furthermore, JNJ-63533054 showed no activity at GPR139’s most homologous relative, GPR142 (Dvorak et al., 2015). In addition, JNJ-63533054 has excellent pharmacokinetic parameters after oral dosing in rodents (Dvorak et al., 2015). Therefore, JNJ-63533054 is an excellent candidate to explore the unknown *in vivo* function of central GPR139. Based on the expression of GPR139 in limbic areas, and the fact that its endogenous agonists are precursors for classical neurotransmitters well known to be involved in mood and anxiety, and that the GPR139 expressing regions have direct and indirect connections with catecholaminergic and serotoninergic nuclei (Phillipson and Pycock, 1982; Bianco and Wilson, 2009; Viswanath et al., 2013; Ogawa et al., 2014; McLaughlin et al., 2017), we hypothesized that the GPR139 agonist may alter behaviors related to habenula and limbic circuitry or interact with drugs that modulate serotonin (5-HT), norepinephrine (NE), and dopamine (DA).

Specifically, we examined GPR139 agonist activation of c-fos in the medial habenula, based on previously reported results (Hitchcock et al., 2015), and also measured extracellular levels of 5-HT, NE, and DA in the prefrontal cortex (mPFC), and DA in the nucleus accumbens (NAc) after amphetamine challenge, in awake and freely moving rats, based on the circuitry described above. Finally, we tested the agonist in a panel of behavioral assays commonly thought to be linked to mood circuitry, including assays related to aversive learning, based on previous reports of the medial habenula (Salas et al., 2009; Baldwin et al., 2011; Frahm et al., 2011; Koppensteiner et al., 2017; Molas et al., 2017), as well as searching for potential interactions with drugs that might interact with the GPR139 system.

## Materials and Methods

### Subjects

Adult, male mice (C57BL/6, Charles River Laboratories, 8–12 weeks upon arrival) and adult, male rats [Sprague–Dawley or Wistar–Kyoto (WKY), see below, Charles River Laboratories, 250–350 g at time of testing] were maintained on a normal 12 h light:dark cycle with food and water provided *ad libitum*. All testing was conducted during the light cycle. Except during behavioral testing, animals were given free access to food and water. All the studies have been carried out in accordance with the *Guide for the Care and Use of Laboratory Animals* as adopted and promulgated by the National Institutes of Health and all protocols have been approved by the local Institutional Animal Care and Use Committee.

### Drugs

For the mouse blood–brain barrier penetration study and c-fos expression studies, JNJ-63533054 was solubilized in 0.5% hydroxypropyl methylcellulose (HPMC) in water at either 1 or 3 mg/ml for oral dosing at 10 ml/kg. Amphetamine (Sigma, St. Louis, MO, United States) was dissolved in saline at 1 mg/ml for i.p. dosing at 2 ml/kg.

For the rat blood–brain barrier penetration study and neurochemistry studies, JNJ-63533054 was placed in suspension for oral (p.o.) administration in 0.5% HPMC in water at concentration of 10 mg/ml and delivered at a volume of 1 ml/kg. For the mPFC neurochemistry study, desipramine (Sigma, St. Louis, MO, United States) was formulated for subcutaneous (s.c.) administration in saline at a concentration of 10 mg/ml and delivered at a volume of 1 ml/kg. For the NAc neurochemistry, D-amphetamine sulfate (Sigma, St. Louis, MO, United States) was formulated for s.c. administration in saline at a free base concentration of 0.3 mg/ml and delivered at a volume of 1 ml/kg.

For the mouse behavioral studies, JNJ-63533054 was dosed p.o. at 10 ml/kg, as a suspension in 0.5% HPMC at the appropriate concentration for the dose. Naloxone HCl, morphine sulfate, fluoxetine HCl, and imipramine HCl were purchased from Sigma (St. Louis, MO, United States) and were dissolved in 0.9% saline at an appropriate concentration for dosing at 10 ml/kg. For the rat behavioral studies, JNJ-63533054 was dosed p.o. at 3 ml/kg, as a suspension in 0.5% HPMC at the appropriate concentration for the dose. Diazepam was dosed s.c. at 1 ml/kg and was prepared as a suspension in sterile H_2_O containing one or two drops of Tween 80.

### Blood–Brain Barrier Penetration

Six groups of three male C57BL/6 mice and three groups of two male Sprague–Dawley rats were used. Animals received an oral administration of JNJ-63533054 at a dose of 10 mg/kg. At various time points, animals were euthanized (mouse study: 0.25, 0.5, 1, 2, 4, and 8 h; rat study: 0.5, 2, and 6 h). Brains were removed and homogenized for liquid chromatography/tandem mass spectrometry analysis. Blood samples were collected by cardiac puncture. All blood samples were deproteinized by 1:4 dilution of the sample with acetonitrile with vigorous mixing. Samples were incubated for 5 min and then centrifuged at 14,0000 rpm in a microcentrifuge for 4 min. A Luna C18(2), 2 mm × 50 mm, 5 U analytical column was used for separation.

### c-fos

Mice were orally dosed with 10 or 30 mg/kg JNJ-63533054 or 0.5% HPMC vehicle. An additional group was given amphetamine (2 mg/kg, i.p.) as a positive control (*N* = 4 animals per treatment group). One hour after dosing, animals were sacrificed and transcardially perfused with 4% paraformaldehyde (PFA). Brains were removed, placed in cold 4% PFA overnight, and washed with phosphate buffer the following day. Coronal sections containing medial habenula and dorsal striatum were cryosectioned at 50 μm, immunostained for c-fos using a primary c-fos antibody (Millipore, catalog# ABE457; 1:15,000 dilution), and visualized with a secondary antibody for Biotinylated Anti-Rabbit (Vector catalog# BA-1000, developed using NiDAB; 1:1000 dilution), and mounted onto glass slides (NeuroScience Associates, Knoxville, TN, United States). Slides were imaged via brightfield imaging on an upright microscope and the number of c-fos labeled cells within the brain areas of interest was quantified with ImageJ software (U. S. National Institutes of Health, Bethesda, MD, United States).

### Neurochemistry

Male Sprague–Dawley rats were implanted with microdialysis guides aimed at mPFC (AP: 3.2 mm, ML: 0.8 mm, V: 1.0 mm; Paxinos and Watson) and the day before collection, microdialysis probes (4 mm active membrane; Eicom USA, San Diego, CA, United States) were inserted. After baseline collection, animals were injected with JNJ-63533054 (10 mg/kg, p.o.) or vehicle and additional samples were collected. As a positive control to demonstrate alterations in neurotransmitter levels, desipramine (10 mg/kg s.c.) was given at the end of the experiment. Samples were analyzed with HPLC with electrochemical detection (Eicom USA, San Diego, CA, United States) for DA, 5-HT, and NE. In a follow-up experiment, the microdialysis probe (2 mm active membrane; Eicom USA, San Diego, CA, United States) was aimed at the NAc (AP: 1.8 mm, ML: 1.6 mm, V: 5.6 mm; Paxinos and Watson) and 30 min after injection of JNJ-63533054 (10 mg/kg, p.o.), D-amphetamine sulfate (0.3 mg/kg s.c., weight of free base) was given and samples were analyzed for DA.

### Naloxone Conditioning

Mice were habituated to a two-sided, gated shuttle box with distinct cues (either black and white waves or stripes, combined with either lemon or almond scent) with the gate open, allowing exposure to both sides, on day 1 for 15 min. Then, in the morning on days 2–4, with the gate closed, mice were confined to one side of the box, chosen randomly, and conditioned for 30 min to saline control. Several hours later in the afternoon, mice were conditioned for 30 min to JNJ-63533054 (10 mg/kg p.o.) + naloxone (10 mg/kg, i.p., weight of salt), or their respective controls, on the opposite side with the gate closed. Morphine (20 mg/kg, s.c.) was given at the end of the day on days 1–3 (i.e., ∼20 h before each naloxone conditioning). Number of withdrawal jumps was recorded during the last afternoon conditioning by an observer blind to treatment. On day 5, a 15 min preference test, with the gate open, was given. Animal position and LMA was recorded each session via a grid of photobeams using MotoMonitor (Kinder Scientific, San Diego, CA, United States).

### Marble Burying

Mice were co-injected with 0 or 10 mg/kg, p.o. JNJ-63533054 and fluoxetine (0–10 mg/kg, i.p.) and 30 min later placed in a test cage filled 5 cm deep with pine shavings that contained 24 marbles dispersed evenly over the top of the bedding. Number of marbles buried (at least two-thirds of the way) was recorded at the end of the 30 min test by a blinded observer, and LMA during the test was measured via photobeams (MotoMonitor, Kinder Scientific, San Diego, CA, United States).

### Elevated Plus Maze (EPM)

The elevated plus maze (EPM) test was conducted at a contract research facility (Behavioral Pharma Inc., La Jolla, CA, United States) using Sprague–Dawley male rats (200–249 g at purchase, Harlan, Livermore, CA, United States). The EPM is made of aluminum that has two open arms (50 cm × 10 cm) and two closed arms of the same size with walls 40 cm high, elevated 86 cm above the ground. Both arms are made of black Plexiglas. The average illumination level on the open arms was 187 LUX and 100 LUX on the closed arms. At the beginning of the experiment, rats were brought into a holding room directly next to the testing room and allowed to habituate to the environment for 30 min. At the commencement of testing, rats were placed in the center of the maze, facing one of the open arms and observed for 5 min. The maze was equipped with infrared beams and sensors capable of measuring time spent on the open and closed arms, number of open and closed arm entries, and XY ambulations on open and closed arms. The results are expressed as mean ratio of time spent in open arms to total time spent in both open and closed arms, mean ratio of open arm entries to total open and closed arm entries, and mean XY ambulations on both open and closed arms. Rats were treated with one of several doses of JNJ-63533054 (10 mg/kg, 30 mg/kg, or vehicle) and placed on the EPM 30 min after treatment. In addition, diazepam, at 1.5 mg/kg, was used as a positive control (administered 30 min prior to testing).

### Female Urine Sniffing and Tail Suspension Test (TST)

Male mice will sniff the scent of female urine, as it is a natural reward. Male mice were habituated to a cotton swab with no scent for at least 30 min and then injected with 0 or 10 mg/kg, p.o. JNJ-63533054, and 30 min later exposed to two new cotton swabs, one dipped in fresh urine pooled from several female mice, and the other dipped in water. Time spent sniffing the cues was measured over 3 min by a blind observer.

For tail suspension test (TST), mice were co-injected with 0 or 10 mg/kg, p.o. JNJ-63533054 and imipramine (0–10 mg/kg, i.p.) and 30 min later hung inverted by the tail. Time spent immobile over the last 4 min of the 6 min test was automatically scored by video software (VideoTrack Software, Viewpoint, Civrieux, France).

### Learned Helplessness

Wistar-Kyoto rats were exposed to a series of 60 inescapable footshocks (60 shocks of 1.0 mA intensity and 15 s duration, with an average interval of 30 s between shocks) in a shuttle box (Gemini shuttle box, San Diego Instruments, San Diego, CA, United States) with the gate closed for learned helplessness induction. On the next 2 days, animals were given screens for helplessness in a test consisting of 30 escapable shocks (0.3 mA intensity and 20 s in duration or ending with escape, with a light cue and the gate opening preceding the shock by 5 s, and an average inter-trial interval of 60 s), with a fixed ratio requirement of two crossings to escape the shock starting at trial 5. Based on number of escapes over these two tests, animals were divided into helpless or resilient groups. These two groups were then tested on the next day 15 min after treatment with 0 or 10 mg/kg, p.o. JNJ-63533054 in the same manner as the screens. To determine if the GPR139 agonist would affect extinction to helplessness, the helpless animals were re-tested with the same pretreatment over 4 additional days.

To determine if the GPR139 agonist could prevent the development of helplessness, WKY rats were injected with 0 or 10 mg/kg, p.o. JNJ-63533054 15 min before the induction and tested the next day in a single test of 30 escapable shocks, and no screens or further treatments, as described above.

### Statistics

The number of c-fos labeled cells within a brain area of interest (medial habenula or dorsal striatum) were counted and averaged across three consecutive sections through that region for each animal to obtain the final values for that animal. C-fos expression was analyzed via a one-way analysis of variance (ANOVA) for treatment. Extracellular levels of the neurotransmitters from the microdialysis experiment were converted into %baseline values, based on the average baseline level, and then analyzed with a two-way ANOVA for treatment × time, with repeated measures on time. Number of jumps observed during the naloxone conditioning was analyzed with a non-parametric Kruskal–Wallis ANOVA. LMA during the three conditioning sessions was measured as total distance traveled (cm) during the conditioning session with a three-way ANOVA for pretreatment × treatment × time, with repeated measures on time. Naloxone place aversion was analyzed as %time spent in the PM chamber with a three-way ANOVA for pretreatment × treatment × test, with repeated measures on test. Number of marbles buried was analyzed with a two way ANOVA for both treatments. EPM was analyzed as %open arm time with a one-way ANOVA for treatment. Urine sniffing was analyzed for time spent sniffing each cue (s) using a two-way ANOVA for treatment × cue, with repeated measures on cue. Tail suspension data were analyzed as time immobile (s) during the last 4 min of the test using a two-way ANOVA for both treatments. Learned helpless data from single tests were analyzed as number of escapes using a one-way ANOVA for treatment, or a two-way ANOVA for treatment × test, with repeated measures on test, when analyzing extinction. ANOVA’s were followed by Duncan’s *post hoc* test when appropriate. All data were analyzed with Statistica software (Statsoft, Tulsa, OK, United States). In all figures, data are presented as mean ± standard error of the mean (SEM). In the blood–brain barrier penetration studies presented in Tables 1, 2, data are presented as mean ± SD.

**Table 1:** Plasma and brain levels of JNJ-63533054 measured at different time points after an oral administration (10 mg/kg) in male mice.

Time (h)	Plasma concentration (ng/mL)	Brain concentration (ng/mL)
0.25	2193 ± 479	2115 ± 585
0.5	1967 ± 250	2106 ± 487
1	1283 ± 150	1373 ± 204
2	508 ± 244	450 ± 156
4	227 ± 115	216 ± 96
8	62 ± 46	45 ± 14

*Data are mean ±SD (n = 3).*

**Table 2:** Plasma and brain levels of JNJ-63533054 measured at different time points after an oral administration (10 mg/kg) in male rats.

Time (h)	Plasma concentration (ng/mL)	Brain concentration (ng/mL)
0.5	350 ± 2.5	416 ± 1.4
2	183 ± 69	217 ± 56
6	81 ± 9	93 ± 10

*Data are mean ±SD (n = 2).*

The experimental details for all the experiments are summarized in Supplementary Table S1.

## Results

### Blood–Brain Barrier Penetration Studies

The blood–brain barrier penetration results are shown in Table 1 (mouse study) and Table 2 (rat study). The compound crossed the blood–brain barrier in both species and the brain to plasma ratio was close to 1 in mouse and slightly higher in rat (1.1–1.2). Absorption was fast as reflected by the highest concentration measured at the first time point (15 min in mouse and 30 min in rat). After administration of an oral dose of 10 mg/kg the maximal brain exposure measured in mouse was 2115 (∼6.7 μM) and 416 ng/mL (∼1.3 μM) in rat.

### C-fos Expression and Neurochemistry

**FIGURE 1:**
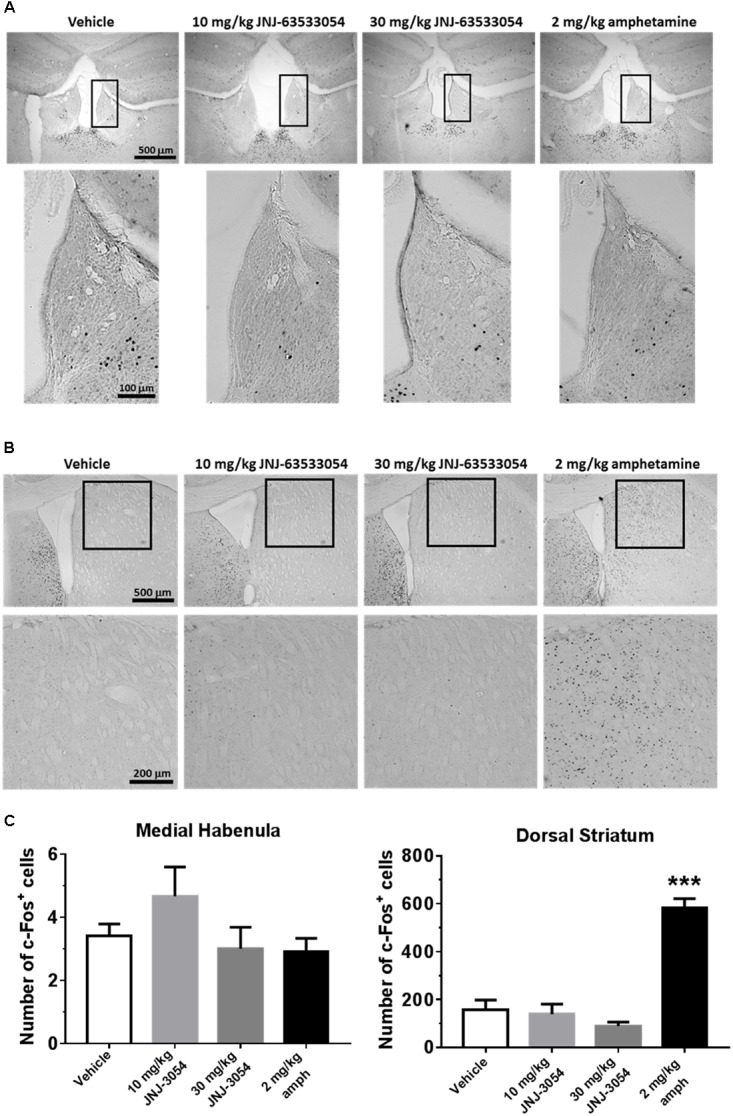
Mouse brain sections immunostained for c-fos expression in medial habenula **(A)** or dorsal striatum **(B)**. Analysis of c-fos expression data (**C**; mean ± SEM). Neither JNJ-63533054 (10 or 30 mg/kg, p.o.) nor amphetamine (2 mg/kg, i.p.) had an effect on c-fos levels in the medial habenula (boxed regions) at 1 h post-injection. As a positive control, amphetamine increased c-fos in dorsal striatum, while JNJ-63533054 was again without effect. ^∗∗∗^*p* < 0.0001; *n* = 4 mice per treatment group.

As GPR139 is highly expressed in the medial habenula and dorsal striatum, we assessed the ability of JNJ-63533054 to stimulate cells in these structures *in vivo* by examining levels of induced c-fos expression. When compared against vehicle-treated control animals, however, JNJ-63533054 had no effect on c-fos expression in the medial habenula (Figure 1A) or dorsal striatum (Figure 1B) at either dose tested (10 and 30 mg/kg, p.o.). As a positive control, amphetamine (2 mg/kg, i.p.) increased c-fos expression in the dorsal striatum but not the medial habenula. Medial habenula: *F*(3,12) = 1.589, *p* = 0.24 for treatment; dorsal striatum: *F*(3,12) = 41.11, *p* < 0.0001 for treatment, with *p* < 0.0001 for amphetamine via *post hoc* Dunnett’s multiple comparison test (Figure 1C).

Using microdialysis in awake, freely moving rats we assessed the effects of JNJ-63533054 administration on DA, 5-HT, and NE levels in the mPFC. The mean basal levels of these neurotransmitters were DA 0.07 ± 0.01 pg/ml, 5-HT 0.06 ± 0.03 pg/ml, and NE 0.31 ± 0.04 pg/ml. All basal means are reported as mean (pg/ml) ± SEM.

**FIGURE 2:**
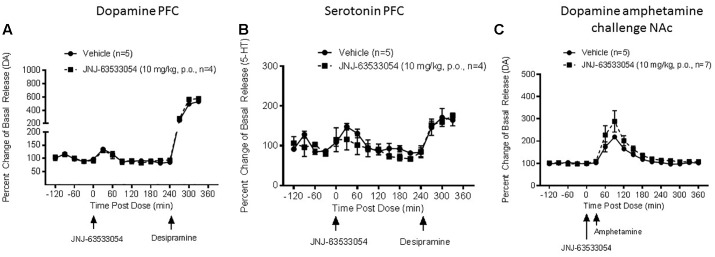
Extracellular levels of DA **(A)** and 5-HT **(B)** in the microdialysate from mPFC in awake and freely moving rats after injection of JNJ-63533054 (10 mg/kg, p.o.) were unaltered, whereas desipramine increased DA and 5-HT as expected. Similarly, JNJ-63533054 did not affect amphetamine (0.3 mg/kg, s.c.)-induced DA in the NAc **(C)**.

JNJ-63533054 (10 mg/kg, p.o.) had no effect of DA, NE, or 5-HT in mPFC [Figure 2A, *F*(12,84) = 0.27, *p* = 0.99 for treatment × time interaction for DA, *N* = 4–5; Figure 2B, *F*(12,84) = 1.15, *p* = 0.33 for treatment × time interaction for 5-HT, *N* = 4–5; and *F*(12,72) = 0.57, *p* = 0.86 for NE, *N* = 3–5, NE data not shown).

As a positive control to demonstrate alterations in neurotransmitter levels in the mPFC, desipramine (10 mg/kg s.c.) was given at the end of the experiment. Desipramine administration increased DA to 530% ± 51 and 575% ± 28, 5-HT 165% ± 15 and 175% ± 7, and NE 322% ± 54 and 235% ± 11 in vehicle and treatment cohorts, respectively. All values reported at mean percent of basal release ± SEM.

In a follow-up microdialysis study, we assessed the effects of JNJ-63533054 administration on DA levels in the NAc. The mean basal level of DA was 0.69 ± 0.07 pg/ml. Basal mean is reported as mean (pg/ml) ± SEM.

JNJ-63533054 had no effect on amphetamine-induced DA release in the NAc [Figure 2C, *F*(1,10) = 1.89, *p* = 0.20 for main effect of pretreatment, *F*(16,160) = 21.56, *p* = 0.0001 for main effect of time, and *F*(16,160) = 1.10, *p* = 0.36 for time × pretreatment interaction, *N* = 5–7].

In the NAc, D-amphetamine sulfate (0.3 mg/kg, s.c.) increased DA to 495% ± 65 and 625% ± 85 in vehicle and treatment cohorts, respectively. Values are reported as mean percent of basal release ± SEM.

### Naloxone Conditioning

**FIGURE 3:**
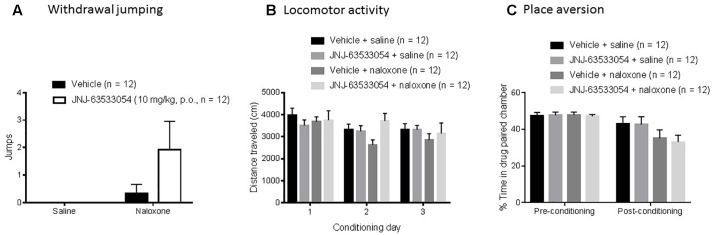
Naloxone (10 mg/kg, i.p.) in sub-chronically morphine (20 mg/kg, s.c.) treated mice induced a significant place aversion **(C)** and a significant increase in withdrawal jumps during the third conditioning **(A)**, but the GPR139 agonist (10 mg/kg, p.o.) had no effects on these measures nor on general locomotor activity during the experiment **(B)**.

Naloxone induced a significant number of withdrawal jumps during the third conditioning [*H*(1,*N* = 48) = 5.45, *p* = 0.02, Kruskal–Wallis ANOVA, naloxone compared to saline]. However, within the naloxone treated group, pretreatment with JNJ-63533054 (10 mg/kg, p.o.) had no effect on number of withdrawal jumps [Figure 3A; *H*(1,*N* = 24) = 2.26, *p* = 0.13, Kruskal–Wallis ANOVA]. In terms of LMA, JNJ-63533054 had no effects throughout the experiment (Figure 3B; *N* = 12 per group, *p* > 0.05, repeated measures ANOVA and Duncan’s *post hoc* tests when appropriate). Naloxone produced a significant place aversion [Figure 3C; *F*(1,44) = 4.75, *p* = 0.03, treatment × test interaction in repeated measures ANOVA with *p* = 0.0075 for naloxone compared to saline in the post-conditioning test, Duncan’s *post hoc*], but JNJ-63533054 did not affect naloxone aversion or produce any preference or aversion on its own (*p* > 0.05 for main effect of GPR139 agonist and interaction in the ANOVA).

### Marble Burying

**FIGURE 4:**
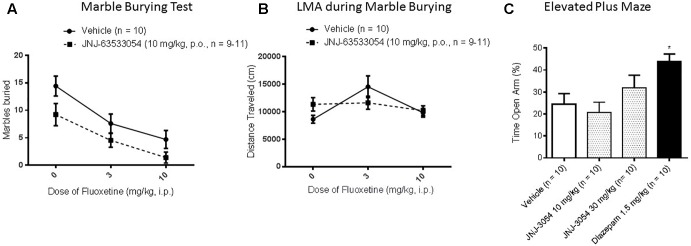
In the mouse marble burying test (mouse) **(A)**, there was a main effect (*p* < 0.05) of GPR139 treatment (10 mg/kg, p.o.) and a main effect of fluoxetine treatment (3–10 mg/kg, p.o.), but no interactions, where both treatments caused a decrease. The GPR139 agonist had no effect on mouse general locomotor activity during the test **(B)**. In EPM performed in rat, the GPR139 agonist (10–30 mg/kg, p.o.) had no effect, whereas the positive control diazepam (1.5 mg/kg s.c.) worked as expected **(C)**. ^∗^*p* < 0.05.

In the marble burying test, a two-way ANOVA revealed a main effect of both treatments [Figure 4A; *F*(2,54) = 15.11, *p* = 0.00001 for main effect of fluoxetine, with *p* < 0.0006, Duncan’s *post hoc*, for both doses of fluoxetine compared to saline, and *F*(1,54) = 8.70, *p* = 0.0047 for main effect of the GPR139 agonist; *N* = 9–11 per group]; however, the decrease in marbles buried produced by JNJ-63533054 (10 mg/kg, p.o.) was only about half the magnitude produced by the positive control fluoxetine (10 mg/kg). Furthermore, there was no interaction between the GPR139 agonist and fluoxetine [*F*(2,54) = 0.26, *p* = 0.77]. The GPR139 agonist had no effects on LMA during the test (Figure 4B; *p* > 0.05 in the two-way ANOVA for total distance for both main effect of GPR139 treatment and interaction). There was an effect of fluoxetine, where 3 mg/kg fluoxetine increased general LMA during the test [*F*(2,54) = 4.44, *p* = 0.016 for main effect of fluoxetine treatment; *p* = 0.02 for 3 mg/kg versus saline, Duncan’s *post hoc*].

### Elevated Plus Maze

In the EPM test, only the positive control diazepam (1.5 mg/kg, s.c.) produced a significant effect, the expected increase in open arm time [Figure 4C; *F*(3,36) = 4.60, *p* = 0.008, *p* < 0.05, Duncan’s *post hoc*, *N* = 10 per group].

### Female Urine Sniffing and Tail Suspension Test

**FIGURE 5:**
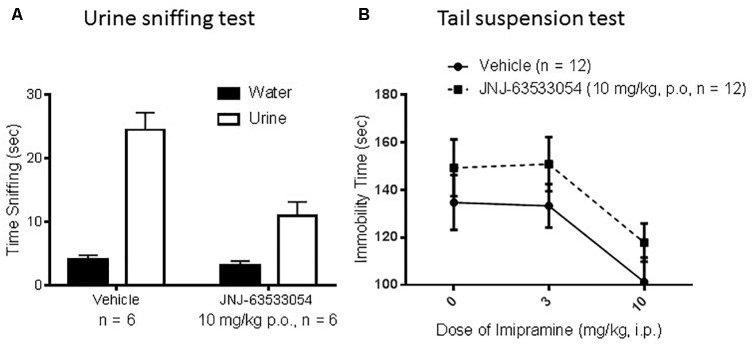
The GPR139 agonist (10 mg/kg, p.o.) produced a significant decrease in time spent sniffing the rewarding mouse female urine cue, but had no effect on time spent sniffing the control cue **(A)**. In mouse tail suspension **(B)**, imipramine (3–10 mg/kg, i.p.) decreased immobility time as expected, but there were no significant effects of the GPR139 agonist (10 mg/kg, p.o.).

In the female urine sniffing test, a two-way ANOVA for cue and treatment, with repeated measures on cue, revealed a significant interaction [Figure 5A; *F*(1,10) = 13.66, *p* = 0.004; *N* = 6 per group]. Duncan *post hoc* tests revealed that JNJ-63533054 (10 mg/kg, p.o.) significantly decreased time spent sniffing the urine cue (*p* = 0.00018) but not the control cue. In the TST (Figure 5B), there was a main effect of imipramine treatment [*F*(2,66) = 6.37, *p* = 0.003] where imipramine decreased immobility time as expected (*p* = 0.003, Duncan’s *post hoc* test for 10 mg/kg). However, the GPR139 agonist had no effect by itself [*F*(1,66) = 3.60, *p* = 0.06] or interaction with imipramine [*F*(2,66) = 0.01, *p* = 0.99; *N* = 12 per group].

### Learned Helplessness

**FIGURE 6:**
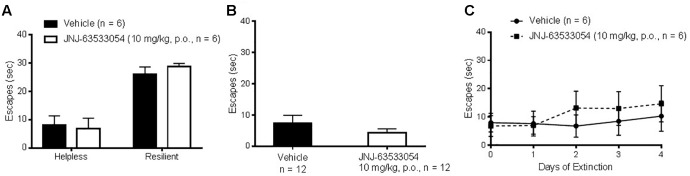
The GPR139 agonist (10 mg/kg, p.o.) dosed either before the test in animals screened for helplessness **(A)** or before the induction **(B)** had no effects on number of escapes. Likewise, sub-chronic treatment with the GPR139 agonist (10 mg/kg, p.o.) in helpless rats had no effects on the rate of extinction **(C)**.

Wistar–Kyoto rats were induced with a series of inescapable shocks, then screened for susceptibility to helplessness, to determine if JNJ-63533054 would affect the number of escapes in either stress-resilient or stress-susceptible animals. As shown in Figure 6A, JNJ-63533054 had no effect in either group [*F*(1,20) = 0.07, *p* = 0.80, main effect of treatment, and *F*(1,20) = 0.45 *p* = 0.51, interaction between treatment and stress group; *N* = 6 per group]. Likewise, when JNJ-63533054 was dosed solely before the shock induction, it did not prevent, nor augment, the development of helplessness [Figure 6B; *F*(1,22) = 1.10, *p* = 0.31; *N* = 12 per group]. Animals that were determined to be helpless in the screen from the first experiment were then repeatedly tested with the same treatment each day to determine if the GPR139 agonist would affect extinction rate. However, there were no effects of treatment on number of escapes [Figure 6C; *F*(1,10) = 0.16, *p* = 0.70 for main effect of treatment and *F*(5,50) = 0.89, *p* = 0.49 for interaction between treatment and test day], only an effect of extinction [*F*(5,50) = 3.91, *p* = 0.0046 for main effect of the repeated measures; *p* < 0.009 for the last three extinction tests versus pre-test day, Duncan’s *post hoc*].

## Discussion

The medial habenula has a high expression of GPR139 receptors (Liu et al., 2015). It has previously been reported that treatment with a GPR139 agonist induced c-fos expression in the medial habenula (Hitchcock et al., 2015). However, our results did not confirm this finding using very similar methods in the same species, perhaps alluding to the complex, still unraveling pharmacology of GPR139, or perhaps due to unknown variables, such as fed state of the animal or housing conditions, that could potentially have an interaction. The blood–brain barrier study demonstrated that the compound is quickly absorbed, crossed the blood–brain barrier, and remained in the brain at relatively high concentration for several hours. The c-fos study was performed at 10 and 30 mg/kg doses achieving a maximal brain concentration of 6.7 and 20μM, respectively (assuming linear pharmacokinetic from 10 to 30 mg/kg). These concentrations were over a 100-fold in excess of the *in vitro* potency value (EC_50_ = 16 nM) measured in an *in vitro* functional assay (Liu et al., 2015). However, it is possible that we missed the c-fos peak activation and selected a time point (60 min) preceding the peak of activation or that repeated administrations are needed to unravel an effect on cfos activation.

As mentioned earlier, the endogenous agonists for GPR139 are precursors for classical neurotransmitters and GPR139 expressing regions have direct and indirect connections with catecholaminergic and serotoninergic nuclei. Therefore, we hypothesized that the GPR139 agonist may alter levels of catecholamines or serotonin. However, the agonist had no effect on basal DA or 5-HT in the PFC, or NAc (unpublished preliminary findings), and no effect on stimulated release. Furthermore, the GPR139 agonist had no interactions with catecholamine/serotonin uptake blockers in the behavioral assays used in this study. It is especially interesting that there was no effect on amphetamine stimulated DA release in rats, as Takeda used modulation of amphetamine stimulated DA release as a main end point after dosing GPR139 agonist in humans (NCT02959892), although the results of that study are unknown at this time.

The medial habenula also has a very high level of opioid receptors (Gardon et al., 2014) and has been implicated in aversion, withdrawal, and negative affect (Salas et al., 2009; Baldwin et al., 2011; Frahm et al., 2011; Koppensteiner et al., 2017; Molas et al., 2017). However, in our studies, pretreatment with the GPR139 agonist before naloxone conditioning in sub-chronically morphine treated rats had no effect on physical withdrawal or the negative affect produced by withdrawal as measured by place aversion. Interestingly, the GPR139 agonist also did not alter place preference in the saline conditioned animals, indicating that the agonist by itself lacks strong interoceptive stimuli. While previous reports implicated the medial habenula in the place aversion effects of nicotine (Frahm et al., 2011), more recent studies also show an involvement of this brain area in the place preference effects of cocaine (Lopez et al., 2018). Thus, one hypothesis may be that the medial habenula is more involved in the motivational value of drugs of abuse, rewarding or aversive, and not just place aversion in general. This would be consistent with a recent report demonstrating modulation of alcohol intake in dependent rats by intra-habenular dosing of the GPR139 agonist (Kononoff et al., 2018).

In the classical tests of anxiety, the GPR139 agonist produced a small decrease in the number of marbles buried, but this possible anxiolytic-like effect did not extend to the EPM. Apart from the intrinsic differences between the two tests (one test measuring xenophobia or compulsion, and the other test measuring fear of open spaces and heights) and methods used (mice versus rats), it is likely the effect observed in the marble burying test was too weak to be seen consistently across models. In the female urine sniffing test, male rodents will spend time sniffing a cue associated with the scent of female urine, as it is a natural reinforcer (Malkesman et al., 2010). In this test, the GPR139 agonist decreased time spent sniffing the urine cue, a possible anhedonic-like effect. This result may be consistent with recent reports that altered medial habenula signaling results in anhedonic-like behaviors, as measured by sucrose preference (Hsu et al., 2014; Han et al., 2017). Although time spent sniffing the control cue was unaltered, total time sniffing the control cue was low and could be subjected to flooring effects; however, general LMA was not affected in any of the other tests by the GPR139 agonist in mouse. Noteworthily, in a previous study in rat the GPR139 agonist was found to decrease spontaneous LMA (Liu et al., 2015) suggesting possible speciation in GPR139 physiology (*in vitro* JNJ-63533054 activates rat and mouse GPR139 with similar potency). A possible anhedonic-like effect could be consistent with the increase in immobility in tail suspension, although that increase failed to reach significance and there were no effects in the learned helpless model, another model related to mood and despair. After testing baseline responses in learned helplessness and finding no effects of GPR139 agonist treatment, we extended the study to look at extinction. The medial habenula and lateral striatum, areas of high GPR139 expression, are known to be involved in extinction, especially to aversive memories such as fear and foot shock (Kobayashi et al., 2013; Zhang et al., 2016; Koppensteiner et al., 2017; Goodman and Packard, 2018). However, again the GPR139 agonist had no effect on rate of extinction in this test.

## Conclusion

In summary, the GPR139 agonist produced a mostly silent signal in these particular *in vivo* models related to mood, with the obvious limitations of current animal models of mood acknowledged. It was recently reported that JNJ-63533054 decreased alcohol intake only in dependent rats showing escalated responses. Thus, GPR139 may be involved in adaptive or habituative functions in response to a chronic state or stimulus, or perhaps the effects of GPR139 activation *in vivo* are only revealed after such ongoing processes alter GPR139 function or expression level. Given this, it would be interesting to test the GPR139 agonist in models of mood and anxiety that have a more chronic component in lieu of the acute or sub-chronic models used here, such as the chronic mild stress model. Similarly, the neurochemistry experiments presented in this manuscript should be repeated under stimulated conditions. Another possible interpretation is that the endogenous signaling level of GPR139 is high, causing agonists to appear mostly silent *in vivo*. Future work exploring the effects of GPR139 antagonists, when available, is needed to further unravel the physiological function of GPR139.

## Author Contributions

JS, TL, CL, and PB participated in research design. NW, IF, RW, and BL conducted the experiments. JS, IF, and RW performed data analysis. JS and PB contributed to the writing of the manuscript.

## Conflict of Interest Statement

All authors are paid employees at Janssen Research & Development, LLC.
