# Supplementary material for: In vivo Characterization of a Selective, Orally Available, and Brain Penetrant Small Molecule GPR139 Agonist
Source: Front Pharmacol. 2019 Mar 21;10:273. doi: 10.3389/fphar.2019.00273 (PMC6437111; doi:10.3389/fphar.2019.00273)
Supplement: Supplementary file 1 [file Table_1.docx]

| Experiment | Species | Experimental groups (all naïve at start) | Timeline | Statistics |
| --- | --- | --- | --- | --- |
| Blood brain barrier penetration | Mouse: C57/BL6  Rat: Sprague Dawley | 1. JNJ-63533054 (10 mg/kg) | 0.25, 0.5, 1,2, 4, 8 h: samples collected in mouse  0.5, 2, 6 h: samples collected in rat | NA |
| c-Fos | C57/BL6 | 1. 10 mg/kg JNJ-63533054  2. 30 mg/kg JNJ-63533054  3. Vehicle  4. 2 mg/kg Amphetamine (c-fos positive control) | 1 h: Samples collected | Medial habenula: No effect of treatment on c-Fos^+^ cell number (F(3, 12)=1.589, p=0.24)  Dorsal striatum:  Significant effect of amphetamine treatment only (F(3, 12)=41.11, p<0.0001) |
| Microdialysis | Sprague Dawley | PFC:  1. JNJ-63533054 (10 mg/kg) + Desipramine (10 mg/kg)  2. Vehicle + Desipramine (10 mg/kg)  NAC:  3. JNJ-63533054 (10 mg/kg) + Amphetamine  4. Vehicle + Desipramine (10 mg/kg) | PFC:  0 h: Baseline started  2 h: Experimental treatment injected  6 h: Desipramine injected  7.5 h: Collection terminated  NAC:  0 h: Baseline started  2 h: Experimental treatment injected  2.5 h: Amphetamine injected  8 h: Collection terminated | PFC:  No effect of JNJ-63533054  (F(12, 84)=0.27, p=0.99)  NAC:  No effect of JNJ-63533054 (F(16, 160)=1.10, p=0.36) |
| Naloxone conditioning | C57/BL6 | 1. Morphine (20 mg/kg) + JNJ-63533054 (10 mg/kg) + Naloxone (10 mg/kg)  2. Morphine (20 mg/kg) + Vehicle + Naloxone (10 mg/kg)  3. Morphine (20 mg/kg) + JNJ-63533054 (10 mg/kg) + Saline  4. Morphine (20 mg/kg) + Vehicle + Saline | Day 1 afternoon: Habituation  Day 1 evening: Morphine injection  Day 2 - 4 morning: Saline conditioning  Day 2 – 4 afternoon: JNJ-63533054 + Naloxone conditioning  Day 2 – 3 evening: Morphine injection  Day 5 afternoon: Test | No effect of JNJ-63533054 on withdrawal (H(1, N=24)=2.26, p=0.13) or conditioning (F(1, 44)=4.75, p>0.05 for post-hoc) |
| Marble burying | C57/BL6 | 1. JNJ-63533054 (10 mg/kg) + 10 mg/kg fluoxetine  2. JNJ-63533054 (10 mg/kg) + 3 mg/kg fluoxetine  3. JNJ-63533054 (10 mg/kg) + saline  4. Vehicle + 10 mg/kg fluoxetine  5. Vehicle + 3 mg/kg fluoxetine  6. Vehicle + saline | 0 h: Co-injection of treatments  0.5 h: Start of marble burying test  1 h: End of test | Minor decrease in marbles buried (F(1, 54)=8.70, p=0.0047) |
| EPM | Sprague Dawley | 1. JNJ-63533054 (30 mg/kg)  2. JNJ-63533054 (10 mg/kg)  3. Vehicle  4. Diazepam | 0 min: injection of treatments  30 min: Start of EPM for 5 min | No effect of JNJ-63533054 (F(3, 36)=4.60, p>0.05 for post-hoc) |
| Urine sniffing | C57/BL6 | 1. JNJ-63533054 (10 mg/kg)  2. Vehicle | 0 min: Habituation  30 min: Injection  60 min: Test started  63 min: Test ended | Decreased urine sniffing (F(1, 10)=13.66, p=0.00018) |
| Tail suspension | C57/BL6 | 1. JNJ-63533054 (10 mg/kg) + 10 mg/kg imipramine  2. JNJ-63533054 (10 mg/kg) + 3 mg/kg imipramine  3. JNJ-63533054 (10 mg/kg) +saline  4. Vehicle + 10 mg/kg imipramine  5. Vehicle + 3 mg/kg imipramine  6. Vehicle + saline | 0 min: Co-injection  30 min: Test started  36 min: Test ended | No effect of JNJ-63533054 (F(1, 66)=3.60, p=0.06) |
| Learned helpless | Wistar Kyoto | Reversal:  1. JNJ-63533054 (10 mg/kg)  2. Vehicle  Prevention:  3. JNJ-63533054 (10 mg/kg)  4. Vehicle | Reversal:  Day 1: Inescapable shocks  Day 2-3: Screen  Day 4 0 min: Injection  Day 4 15 min: Test started  Day 4 ~60 min: Test ended  Day 5 -8: Repeat of Day 4  Prevention:  Day 1 0 min: Injection  Day 1 15 min: Inescapable shocks started  Day 2: Test | Reversal:  No effect of JNJ-63533054 on test day (F(1, 20)=0.45 p=0.51) or extinction (F(5, 50)=0.89, p=0.49)  Prevention:  No effect of JNJ-63533054 (F(1, 22)=1.10, p=0.31) |

Supplemental table summarizing the experimental details.
